# Testing the Novel Weapons Hypothesis of the Argentine Ant Venom on Amphibians

**DOI:** 10.3390/toxins15040235

**Published:** 2023-03-23

**Authors:** Juan Pablo Llopart, Paloma Alvarez-Blanco, Lucía Moreira-Demarco, Alok Bang, Elena Angulo, Raúl Maneyro

**Affiliations:** 1 Laboratorio de Sistemática e Historia Natural de Vertebrados, Facultad de Ciencias, Universidad de la República, Montevideo 11400, Uruguay; 2 Estación Biológica de Doñana (CSIC), Av. Américo Vespucio 26, 41092 Sevilla, Spain; 3 School of Arts and Sciences, Azim Premji University, Bangalore 562125, India; 4 Society for Ecology Evolution and Development, Wardha 442001, India

**Keywords:** chemical weapon, invasive species, *Linepithema humile*, predator-prey relationships, toxic dose, amphibian decline, iridomyrmecin

## Abstract

The globally invasive Argentine ant (*Linepithema humile*) possesses a venom lethal to some amphibian species in the invaded range. To test the novel weapons hypothesis (NWH), the effects of the toxin on the cohabiting amphibian species in the ant’s native range need to be investigated. The invader should benefit from the novel chemical in the invaded range, because the species are not adapted, but the venom should not be effective in the native range. We explore the venom effects on juveniles of three amphibian species with different degrees of myrmecophagy inhabiting the ant’s native range: *Rhinella arenarum*, *Odontophrynus americanus,* and *Boana pulchella*. We exposed the amphibians to the ant venom, determined the toxic dose, and evaluated the short- (10 min to 24 h) and medium-term (14 days) effects. All amphibian species were affected by the venom independently of myrmecophagy. In addition to amphibian sensitivity, we discuss how the differential Argentine ant abundance and density in the two ranges could be the key to the susceptibility of amphibians to the venom, resulting in the possibility of NWH. Our results confirm the potential magnitude of the impact of the Argentine ant in successfully invaded areas for the conservation of already threatened amphibians.

## 1. Introduction

Biological invasions have a massive impact on ecosystems and are one of the leading causes of biodiversity loss [1]. However, several elements can compromise our ability to predict and prevent the impact of invasions on native species [2,3,4,5]. Some effects of invasions are neither predictable nor obvious, such as those affecting ecosystem processes or functioning [6], and the impacts of the invader on the native species are often underestimated [1].

The Argentine ant, *Linepithema humile*, is among the five invasive ant species categorized as the worst invasive species [7], together with the fire ant, *Solenopsis invicta*, the little fire ant, *Wasmannia auropunctata*, the yellow crazy ant, *Anoplolepis gracilipes*, and the African big-headed ant, *Pheidole megacephala*. These ants have common characteristics that make them unique in conquering new environments [8]: most of them are unicolonial, i.e., ants of different nests cooperate [9], have multiple queens per colony (polygyny), are omnivorous, and are highly aggressive against other species [8,10,11]. This last characteristic is intriguingly important for the success of some ant species that use different attack and defense mechanisms [9,11]. Three of these five invasive ants employ chemical defenses: *S. invicta* and *W. auropunctata*, which inject alkaloids via a stinger, and *A. gracilipes* releases formic acid as a spray. Although the Argentine ant does not have a stinger, it sprays a potent venom effectively against competitors and predators in areas it successfully invades [12,13]. Thus, negative impacts caused by the Argentine ant attain higher trophic levels, such as on native predators [14,15,16]. Outside the predator-prey relationships, the Argentine ant has also been shown as a stressful factor in the breeding of passerines (see examples in [17]. Moreover, it has been showed the potential of the venom of the Argentine ant, iridomyrmecin, to kill amphibians in the invaded ranges [13].

Ant colonies fulfill fundamental ecological roles in their native ranges [8], such as in food chains, with several species feeding primarily on ants, as is the case of reptiles [18], invertebrates [19], mammals [20], amphibians [21], and birds [22]. Postmetamorphic anuran amphibians are mainly carnivorous [23], and some species have specialized diets towards myrmecophagy [24]. This selection is favored in some groups of amphibians by taking alkaloids present in the ants, acquiring unique characteristics such as the secretion of alkaloids through the skin and becoming toxic depending on the abundance and species of ants consumed in the diet [24,25]. This anti-predator tactic could have exerted selective pressure on these groups [24]. 

When an invasive prey species occupies a new ecosystem, native predators have not had the necessary co-evolutionary time, so they do not have mechanisms that allow them to deal with the toxins used by these new prey [26,27]. Moreover, invasive species with chemical weapons can have a high invasion success if the weapon allows them to face the resident species that cannot deal with them [28]. Called the novel weapons hypothesis (NWH), it has been demonstrated with plants presenting allopatric chemicals that inhibit potential competitors in the invaded ranges; native species of invaded ranges are not adapted to the novel weapon, which enhances the invader’s competitive ability and success [28,29]. 

Here, we propose that this hypothesis could explain the successful invasions of *L. humile* since its venom can be lethal in juvenile amphibians in the areas successfully invaded [13]. The novel weapons hypothesis assumes that the weapon exists in the species in its native range but does not impact co-existing species; one mechanism to explain this fact is co-evolution, such that species in the native range could have co-evolved and adapted to the weapon. In the native range, the relationships of the Argentine ant with its potential predators, or the effects of its toxin on them, is unknown. Thus, we examined the effect of the Argentine ant venom on amphibians co-existing with the ant in its native area. Because amphibians with different degrees of myrmecophagy could have co-evolved and adapted to the prey’s venom differentially [24], we used juveniles of three amphibian species with different myrmecophagy degrees: *Rhinella arenarum* presents a high number of ants in its diet, especially in its juvenile state [30,31]; *Odontophrynus americanus* has an intermediate degree [32,33], and *Boana pulchella* presents the smallest number in its diet [34,35]. We evaluated the venom’s immediate effect after exposure, monitored the short- and medium-term effects, and discussed the results along with the venom impacts in invaded ranges. Our working hypothesis is that the effects of these toxins on native amphibians will: (i) be inversely associated with their degree of myrmecophagy, with lower toxicity with an increased ant-specialized diet of the amphibians, and (ii) present lower toxicity in the amphibian species of the ant native area, if there is co-evolution, than in amphibian species from Europe. Finally, we discuss the factors that could affect the differential exposure and susceptibility of amphibians to the venom in the field, such as the composition of ant communities and abundance of the Argentine ant in the native and invasive ranges, to give insights about how the novel weapons hypothesis could operate in the case of the venom of the Argentine ant. 

## 2. Results

### 2.1. Toxic Doses to the Argentine Ant Venom 

We applied different doses of the Argentine ant venom on the back of the juvenile amphibians for ten minutes, following the same protocol as in [13], in order to know the toxic dose for each of the three amphibian species. Maximum doses were 350 ants/g in *R. arenarum* and 400 ants/g in *O. americanus* and *B. pulchella* to obtain an effect but not an overdose; this means around 80 ants per individual for *R. arenarum*, 356 for *O. americanus,* and 160 for *B. pulchella*.

Venom toxicity was considered by the animals showing neurological damage ten minutes after the application of the venom. An individual was categorized as affected in the clinical evaluation if any of the physiological responses that show neurological damage (pupillary reflex, flexor reflex, pain, motor response, or palpebral reflex) was altered. As in [13], we observed that 10 min after the application of the venom, some of the treated animals showed general paralysis, sometimes accompanied by extraocular paralysis, loss of photopupillary and palpebral reflexes, and loss of nociceptive response (pain response) (File S1).

**Figure 1:**
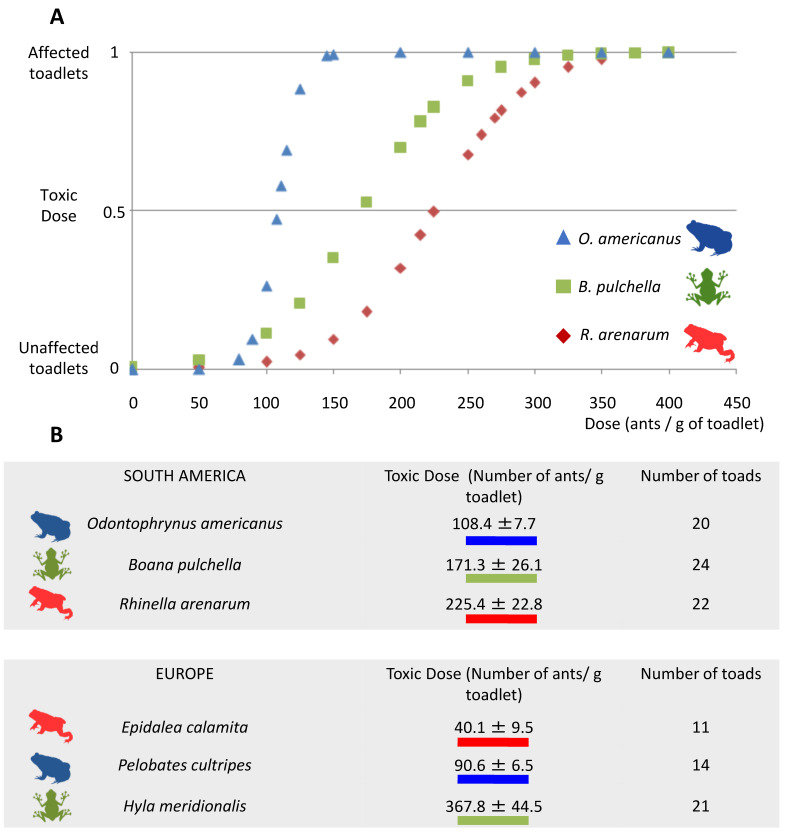
(**A**). Dose-response curves of the toxic effect of the venom of the Argentine ant, *L. humile*, towards juvenile amphibians of three species (*Rhinella arenarum*, *Boana pulchella*, and *Odontophrynus americanus*). (**B**). Mean (±SE) toxic dose of ants that elicited an effect in juvenile amphibians in our study; for comparison, we provide the data modified from Figure 2b in [13], with similar ecological amphibian species in Europe. In (**A**), the Y-label represents the binomial variable of being or not affected by the dose applied. The curves are the dose-response curves that result from calculating the toxic dose for each species using the *dose.p* function in the MASS package in R 3.6.2.

All three amphibian species were negatively affected by exposure to *L. humile* ant venom. *O. americanus* was the most sensitive, with a toxic dose (TD) of 108.4 ± 7.7 ants/g of amphibian, followed by *B. pulchella* (TD = 171.3 ± 26.1 ants/g of amphibian) and *R. arenarum* (TD = 225.4 ± 22.8 ants/g of amphibian) (Figure 1A). Any of the control animals were classified as “affected” in the clinical evaluation. The juveniles of *R. arenarum*, which exhibit the most myrmecophagy, presented the highest toxic dose, while the least myrmecophagous, *B. pulchella,* presented an intermediate dose. *O. americanus*, which has an intermediate degree of myrmecophagy, presented the lowest toxic dose. Toxic doses of the three amphibian species tested had intermediate values compared with toxic doses of the three amphibian species in the invasive range (Figure 1B). 

### 2.2. Short-Term Recovery after Venom Application 

Amphibians followed a clinical evaluation to evaluate their physiological status, which was categorized as affected or unaffected, 10 min, 1 h, and 24 h after applying the toxic doses; mortality was also monitored. 

**Figure 2:**
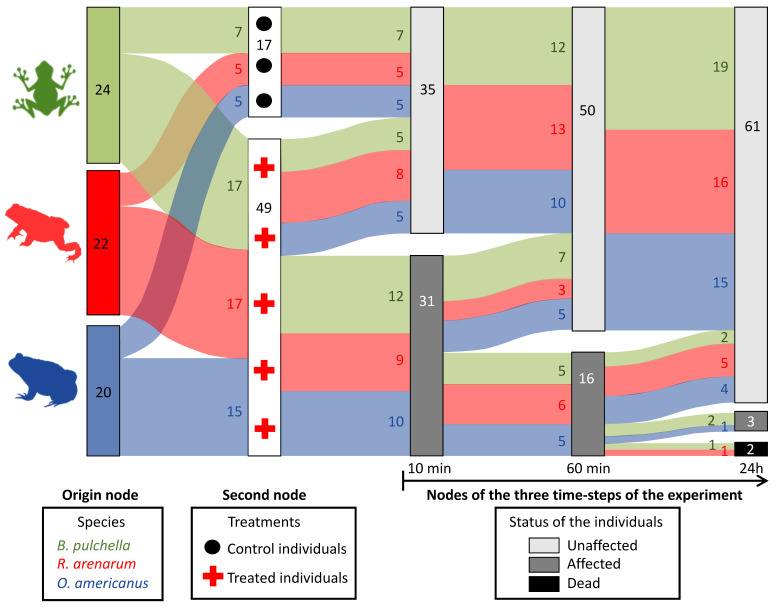
Flow chart showing the evolution of individuals of three amphibian species at 10 min, 60 min, and 24 h after treatment with the Argentine ant venom. The species are shown in the left column. Groups of treated (red crosses) and untreated individuals (control, black dots) are shown in the following column linked by the flow to the first column. The flow of experimental animals along the three main time-steps is shown in the last three columns, where the groups of experimental animals are split or combined by their status: light gray, unaffected individuals; dark gray, affected but living individuals; black, affected dead individuals. An affected individual showed at least one abnormal response in the pupillary reflex, the flexor reflex, the palpebral reflex, the pain response, or the motor response. The sample size is shown inside each column and in the flows. Diagram created with the *sankeyNetwork* function, NetworkD3 package in R 3.6.2 [36].

During this short-term follow-up, only one individual of *R. arenarum* and one of *B. pulchella* died within 24 h, and one individual of *B. pulchella* and one of *O. americanus* were still affected 24 h after applying the venom (Figure 2). During this time, treated individuals of the three species recovered, reducing the number of affected individuals from 31 to 16 and then to 3, after 24 h (Figure 2). The trend regarding the myrmecophagy of each species was not clear: the least ant consumer, *B. pulchella,* was the most affected in 24 h after applying the venom, with one individual dead and another affected. The highest ant consumer, *R. arenarum*, was partially affected, with one individual dead, and *O. americanus* seemed to be the least affected, with only one individual affected after 24 h.

### 2.3. Medium-Term Effects of the Argentine Ant Venom

**Figure 3:**
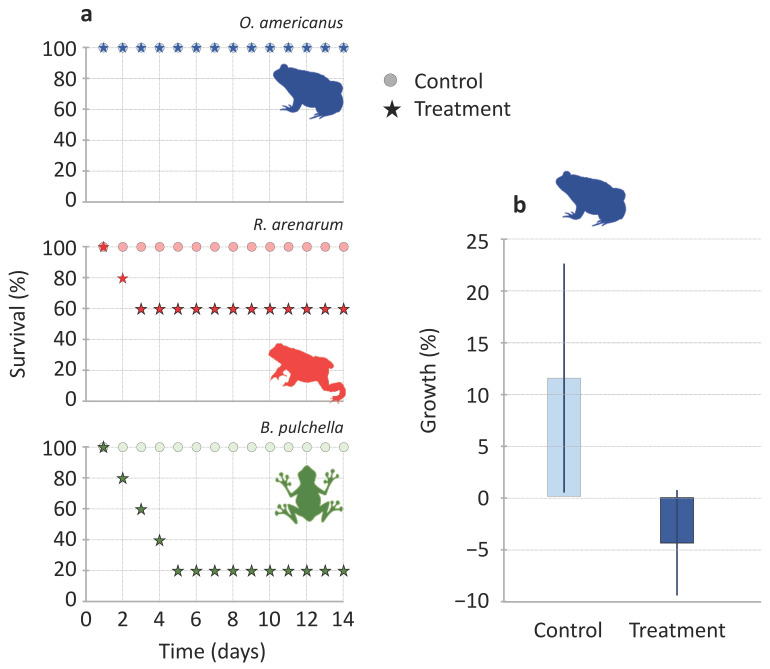
(**a**) Survival along 14 days of juvenile amphibians of three species after exposure to the venom of the Argentine ant, *Linepithema humile*, (stars, *n* = 5), and controls, without exposure to venom (circles, *n* = 5). (**b**) Growth of *Odontophrynus americanus* juveniles during the 14 days of follow-up (mean value ± SE, *n* = 5 control and 5 treated individuals).

Ten individuals of each species (5 control and 5 treated and affected) were monitored at seven and fourteen days to know their medium-term survival and growth. During this medium-term follow-up, the survival of *B. pulchella* was the lowest and decreased consecutively during the first four days to 20% (four individuals died), while *R. arenarum* decreased to 60% in the first two consecutive days (two individuals died, Figure 3a). All individuals of *O. americanus* survived the 14 days (*n* = 5), as well as all the individuals used as controls (*n* = 17, Figure 3a). Survival was significantly different for treated and control individuals in *B. pulchella* (X^2^ = 6.1, *p* = 0.01, *n* = 5) but not in *R. arenarum* (X^2^ = 2.2, *p* = 0.1, *n* = 5) and *O. americanus* in which all individuals survived (*n* = 5). 

In *O. americanus*, control individuals grew up to an average of 11.53 ± 11.10%, while treated individuals lost mass by 4.43 ± 5.12% (Figure 3b). In *R. arenarum*, control individuals grew up to an average of 3.31 ± 5.29% (*n* = 5), while the three surviving individuals lost mass by 9.44 ± 10.05%. In *B. pulchella*, the single survivor had the lowest growth (11.36%) with respect to all the individuals in the control treatment (39.4 ± 20.53%, *n* = 5). Regarding the myrmecophagy degree, the least ant consumer, *B. pulchella*, had the lowest survival at the medium-term. However, both the highest ant consumers, *R. arenarum* and *O. americanus,* also showed medium-term effects after receiving an effective dose of the venom. 

## 3. Discussion

We investigated whether the Argentine ant venom could be considered a novel weapon by checking its impact on the survival of amphibians in the ant’s native range. It has been demonstrated that the venom of the Argentinean ants kills juvenile amphibians in invaded ranges. We hypothesized that amphibians that have co-evolved with the ant in its native range could have adapted to the venom effects, having a higher resistance to the venom than amphibians in the invasive range, and a decreasing effect with increasing amphibian myrmecophagy. Our results show that the venom can kill the amphibian in the ant’s native range, although the effect is not clear regarding the degree of myrmecophagy of the amphibian species or their higher resistance than the amphibians of the invasive range. Because we do not directly demonstrate that venom resistance is related to co-evolution, we propose an alternative mechanism of how the novel weapons hypothesis (NWH) could apply in this case. 

### 3.1. Toxicity to Amphibians in the Argentine Ant’s Native Range

The amphibians tested in our experiments were significantly affected by the ant’s venom, but with different toxic doses. As we predicted, the species with the highest degree of ant specialization, *R. arenarum*, was the least affected by the venom dose. This was the opposite situation to the amphibian species tested in the invasive range, where the most ant-specialized amphibian, *Epidalea calamita*, was the most sensitive [13]. Although the less ant-specialized amphibian had the lowest survival in our experiment, the amphibian species with an intermediate myrmecophagy degree had the highest survival, which does not follow the hypothesis well. A concern could be raised if the species tested, such as *R. arenarum*, do not eat Argentine ants in the field, but other ant species. However, stomach contents of this species in the same locations reveal that they consumed *Solenopsis* and *Linepithema* as the main prey [31]. Furthermore, the three amphibian species selected for this study appear to use the same micro-habitat as the Argentine ant and share part of their distribution ranges (see Appendix S5 in [13]), which makes it likely that the Argentine ant is consumed. 

Regarding co-evolution by the distributional range, amphibians from the native range presented lower toxic dose values than amphibians from the invasive range, except in one species (Figure 1). Again, this result is not consistent with what was predicted. One limitation of this study is that we did not use iridomyrmecin, but the whole body of the Argentine ants, in which other compounds were also present. The use of mashed ants or iridomyrmecin produced the same symptoms, mainly paralysis of individuals [13]. Finally, in [13] and in the present study, the toxic doses are measured as the number of ants per gram of amphibian. 

The toxic dose value for *E. calamita* was almost six times lower than its American counterpart, *R. arenarum*. Both species belong to the Bufonidae Family, and their juveniles are very myrmecophagous [16,31]. In the case of the European species *Pelobates cultripes*, its toxic dose value is significantly lower than that of its American counterpart *O. americanus*, although these species do not pertain to the same Family (Pelobatidae and Odontophrynidae, respectively). Finally, in the Hylidae Family, *Hyla meridionalis* is twice as susceptible to the venom than its American counterpart *B. pulchella*, the latter relationship being opposed to our predictions (Figure 1). It would be interesting to test the venom with amphibians that maintain a formicivore diet as adults, such as *Elachistocleis bicolor* [21] or *Melanophryniscus devincenzii* [37].

We examined the fate of the venom 14 days after exposure, while [13] only surveyed individual recovery during 48 h. Previous works [13] assumed that paralysis was equivalent to death for juveniles because it would have occurred if the juvenile remained in the invaded Argentine ant area. In fact, in laboratory experiments introducing juvenile amphibians in the arena of artificial Argentine ant nests, reported that the metamorphic died if it was not removed 1 min after paralysis began, very likely because of an overdose [13]. In the native area of the Argentine ant, death is less likely because the abundance of the Argentine ant is lower (see Section 3.2); however, temporal paralysis will decrease survival probability because, for example, it limits the capacity of individuals to search for cover against other predators or desiccation, or prevents foraging. A previous work showed that a proportion of affected individuals recovered within 48 h [13]. Our monitoring showed the importance of medium-term recovery in native areas: 40% of *R. arenarum* and 80% of *B. pulchella* died in the following days after exposure, while *O. americanus* survived the 14 days of monitoring. *Odontophrynus americanus* survival may be related to its tetraploid genome; redundancy in its genetic material can allow a better response to cytotoxins [38]. Amphibian behavior also affects the effectiveness of the venom: juveniles of *H. meridionalis* could escape in the field after two minutes in contact with an Argentine ant trail [13]. This could also be the case for *B. pulchella*, especially because Argentine ant abundance or density is low, for example, in the wetlands and forest near the Paraná River (Buenos Aires, Argentine; [39]).

### 3.2. The Importance of Ant Community in Exposure to the Venom

The NWH [28] was initially proposed in plants indicating that certain invasive species bring with them a set of chemicals that are relatively ineffective against species in their native communities but are highly inhibitory against species in the invaded community, which are primarily naive to these products. It has also been demonstrated in some animal systems, such as birds, but in this case, it is mediated by the introduction of new parasites to the invaded bird community [40,41]. To verify the NWH, it is necessary to compare the effects produced by the novel weapon in the native and invasive range [42]. However, the results obtained in the laboratory cannot be linearly extrapolated to what occurs in nature; the scenario is more complex in the field.

The NWH assumes that the weapon exists in the species in its native range but that co-occurring species are not affected, for example, if they have co-evolved and adapted to the weapon [28]. Alternatively, the relationship between amphibians and ants relies on the differential availability of prey (Argentine ants) and ant community compositions between the invasive and native ranges. In the native range, *Linepithema* is neither in great abundance, nor the only ant species; instead, its abundance is mainly limited by competition in the native ant community [43,44,45]. Because *Linepithema* is not the only available species for formicivores [31], the possible co-evolution of amphibians in the native range to the Argentine ant venom is unlikely. In the invasive range, *L. humile* replaces most native ant species, becoming the only ant prey present and in very high abundance [46,47]. This could favor specialist ant predators, as it occurs in Japan with an ant-specialist spider [15]. However, it could also negatively affect ant predators, such as in California, where the horned lizard abundance declined due to the homogenization of the ant community [14]. In a successfully invaded ant community, the amphibians that emerge from the ponds are defenseless against the toxin: [13] assumed that “affected” individuals were equivalent to dead individuals, because a paralyzed individual in Argentine ant-invaded areas will be finally predated by the ants, due to their high density and abundance. Thus, the NWH works because ant availability is different in both ranges: amphibians of the invasive range are exposed to the venom in the field, while the venom is ineffective in amphibians of the native range (where Argentine ants are scarce, less contact in the field = lower lethality in the field = no co-evolution). 

## 4. Conclusions

Our results have implications for amphibian conservation in invaded areas. Venom toxicity of the Argentine ant in the three species tested here is proof of its high unspecificity. Together with the three species tested by [13], this study shows the potential magnitude of the negative impacts of this novel weapon for already threatened taxa, the amphibians. As was predicted [13], more than 800 amphibian species overlap with the Argentine ant in the invasive range worldwide, from which 6.2% are classified as threatened species (IUCN Red List). Thus, looking at the global spread of the invasive Argentine ant [48], locations with threatened amphibians must be highlighted and prioritized for management, as the impact of the invader should not be underestimated.

## 5. Materials and Methods

### 5.1. Collection of Species and Housing

In October 2017, we collected specimens of three amphibian species in Uruguay: *Rhinella arenarum*, *Odontophrynus americanus*, and *Boana pulchella*. Specimens of *R. arenarum* were collected in the locality of Shangrilá (34°52′ S, 56°0′ W), those of *O. americanus* in “El Pinar” (34°48′ S, 55°54′ W), both in the department of Canelones, and those of *B. pulchella* in the surroundings of Melilla, department of Montevideo (34°26′ S, 56°23′ W), Uruguay (File S1). They were collected in the larval stage and reared in the laboratory until metamorphosis, after which they were placed in individual conditioned containers. Tadpoles were fed ad libitum with boiled lettuce and reared in artificially aerated aquariums.

Juveniles of *O. americanus* and *R. arenarum* were housed in a sandy substrate with a shelter and fed with workers of the termite *Cortaritermes fulviceps* (we did not use termite soldiers to avoid bias with the effects of the poison they present). We used a sandy substrate (fine sand from the collection site) for two of the species, which is allowed when maintaining terrestrial amphibians in laboratory conditions, especially if they bury; we kept the humidity high by spraying water inside the containers. *Boana pulchella* juveniles were conditioned with moist soil and branches for environmental enrichment and were fed with *Drosophila melanogaster* flies due to their diet preferences [34]. All amphibians used in the experiment were metamorphic juveniles and were weighed before the experiments (as weight was used to calculate the toxic doses) and then placed under controlled light conditions (subject to the natural circadian cycle). 

*Linepithema humile* ants were collected from a nest in “Parque Rodó” in Montevideo, Uruguay (File S1) and kept in the laboratory in a container where sugar water and insects were supplied ad libitum.

### 5.2. Dose-Response Experiment

We prepared doses of the toxin as in [13]: Argentine ants (whole-body) were macerated in a ceramic bowl with 0.2 mL of dechlorinated water (the venom is soluble in water and most organic solvents) [49]. Thus, doses consisted of a number of ants per amphibian mass. The bowl and the ant container were close to each other. The ants were taken carefully with flexible tweezers and deposited quietly into the bowl. Because the bowl contained water, it was quick to leave the ant in the water with the rest of the ants. Moreover, previous experiments showed that iridomyrmecin quantities were not significantly altered when the ants were disturbed [50].

Amphibians were of small sizes, as they were metamorphic; at the start of the experiment, their weights were as follows (mean and standard deviation): 0.40 g ± 0.09 for *B. pulchella*, 0.89 g ± 0.18 for *O americanus*, and 0.23 g ± 0.02 for *R. arenarum*. This means that a dose of 100 ants per gram of amphibian will represent around 40 ants for *B. pulchella*, around 89 ants for *O. americanus,* and around 23 ants for *R. arenarum*, depending on the weight of each individual (see data of the dose applied and the weight of individuals in File S1).

The toxin to be tested is iridomyrmecin, which represents 2% of the body mass of these individuals and is very volatile [51], so care must be taken to ensure that amphibians receive the correct dose. To this end, a single dose of the mash (ants/gram of individual amphibian) was immediately applied to the back of one amphibian specimen (treated individuals), without reaching the head, and the individual was left in the dark to minimize handling stress for 10 min, after which the mash was removed with water. Although other compounds besides the venom could be in the mash, [13] showed that the use of mashed ants or iridomyrmecin alone produced the same symptoms, mainly paralysis of individuals. 

We conducted the experiments with 20 individuals of *O. americanus* (5 control, 15 treated), 22 of *R. arenarum* (5 control, 17 treated), and 24 for *B. pulchella* (7 control, 17 treated). Individuals for control and treatments were randomly selected. Treatments contained different numbers of Argentine ants mashed, while controls contained only 0.2 mL of dechlorinated water (see File S1). Each dose was elaborated and applied to the juvenile before starting with another experimental animal. The way in which each dose was selected was predetermined for the first experimental animals of each species (including a low, a medium, and a high dose), then adjusted considering the effects already obtained in order to have the sufficient number of individuals around the supposed toxic dose; this allowed the calculation of the dose-response curve. Control animals were intercalated randomly between treated animals.

We assessed the immediate response of each individual to treatment, with a pre- and post-treatment clinical evaluation to categorize the individual as “affected” or “unaffected” considering different physiological responses [13,52]. We examined five parameters reflective of the functioning of different parts of the neurological system, such as the medulla oblongata and pontine nucleus (the trigeminal [V] and facial [VII] cranial nerves) and the midbrain (the oculomotor cranial nerve [III]). When one or more of these physiological responses were altered, the individual was categorized as “affected”, as any of these measures show neurological damages: (i) pupillary reflex was evaluated with a light stimulus, by provoking the contraction or relaxation of the pupil (the light provokes the contraction of the pupil in unaffected individuals); (ii) flexor reflex was provoked by stretching one of the posterior extremities hoping to see fast contractions in case it was unaffected (there is no resistance to the extension of the tarsus in affected individuals); (iii) superficial nociceptive response (pain) was assessed by lightly pressing one of the individual’s extremities with a clamp, causing a muscle contraction (the extremity will not move in affected individuals); (iv) motor response was evaluated by touching it near the urostyle lightly to see a sequence of consecutive jumps (the individual was touched with a clamp twice, the action was repeated to confirm the response, especially if no response was observed, as in affected individuals); and, (v) the palpebral reflex was evaluated when the eyelid muscles were stimulated by pressing on the lateral edge (left and right) of the eyelid (the eyelid closed when touched in unaffected individuals). Previous experience to perform clinical evaluation was acquired under the presence of a veterinarian (Alejandro Bertó-Moran, see [13]. Responses to the three measures of reflex (pupillary, flexor, and palpebral) were objective; they occur or not. The motor and superficial nociceptive responses were clear when the individuals were affected because they showed general paralysis (such as in Figure 3a in [13]). 

Ten of the twenty individuals were selected for medium-term daily monitoring (14 days). Five were controls, and five affected individuals were assigned as treatments (see File S1). The individuals were monitored daily for survival. On the seventh and fourteenth days, the clinical evaluation was repeated, and the individuals were weighed. All the collected individuals were euthanized at the end of the experiments.

All individuals were handled the same way, going through the same procedure. 

### 5.3. Data Analysis

We first calculated the toxic dose (TD) for each amphibian species, which is defined as the number of ants per amphibian mass expected to cause a toxic effect [13]. It was estimated from the dose-response curves of each amphibian species using the *dose.p* function in the MASS package in R 3.6.2 [53,54].

We then described the short-term effect of the venom, using the clinical evaluations of each juvenile at three-time steps after venom exposure: 10 min, 1 h, and 24 h. We described how the status of each individual changed using a Sankey diagram, which was made through the *sankeyNetwork* function of the NetworkD3 package in R 3.6.2 [36]. A Sankey diagram is a graphic illustration of flows; in this case, the experimental animals of each species treated or not will flow along the three time-steps surveys. The groups of animals move from the left nodes (columns) to the right nodes at each step of time, in which their status can be unaffected, affected, or dead. The origin node on the left describes the three species in which the experimental animals are grouped. The second node describes how they are split when assigned to the treatments (how many animals are treated with the venom and how many are in the control category). Then, the three following nodes represent the number of animals in each status determined by the clinical evaluation. Experimental animals move to unaffected, affected, or dead in each node; the flow represents the number that split or combine in each category between two surveys. Unaffected individuals in the first of these three nodes, such as control animals, should normally remain unaffected until the last survey time. Affected individuals can continue to be affected in the following node, can die, or can recuperate and be unaffected. Dead individuals will remain dead.

The medium-term effect of the Argentine ant venom was analyzed in the 10 selected individuals per amphibian species during the 14 days of follow-up. First, in *R. arenarum* and *B. pulchella*, because some individuals died during the follow-up period, a survival analysis was carried out to estimate the probability of mortality as a function of time (survival package in R 3.6.2 [55]. The survival function of each amphibian species was estimated by the Kaplan–Meier method (*survfit* function), and survival was compared between treated and control individuals using the Mantel–Haenszel test (*survdiff* function). In *O. americanus*, because all the individuals survived, the analysis was not necessary. Second, we measured the percentage of growth of individuals from the start of the experiment to the end of the 14 days. For each species, we compared the differences in the growth of control individuals and treated individuals. The mean (±SE) and percentage of growth was calculated as the difference between the initial and the final mass of each individual.

## Data Availability

The data presented in this study are available in File S1.

## Supplementary Materials

The following supporting information can be downloaded at: https://www.mdpi.com/article/10.3390/toxins15040235/s1. File S1 contains data used in the article. File S1: The data used in this article is separated into six spreadsheets: (1) Global Monitoring: The monitoring of each individual with identification (ID), date of experiments, weight, number of ants applied, dose applied, and its survey of all clinic parameters and the fate for the 10 min, 60 min, 24 h, 7 and 14 days; (2) Sampling Locations: locations for the sampling of amphibian and ant species; (3) Dosis-Resp_10 min: data of the dose experiment; (4) Data_Sankey24 h: monitoring of individuals along 24 h; (5) Survival14 days: a survey of the individual survival along 14 days; (6) Growth14 days: monitoring the growth of Odontophrynus americanus along 14 days.
